# Presepsin values and prognostic nutritional index predict mortality in intensive care unit patients with sepsis: a pilot study

**DOI:** 10.1186/s13104-021-05659-9

**Published:** 2021-06-30

**Authors:** Yuichiro Shimoyama, Osamu Umegaki, Noriko Kadono, Toshiaki Minami

**Affiliations:** 1 Department of Anesthesiology, Osaka Medical College, Intensive Care Unit, Osaka Medical College Hospital, 2-7 Daigaku-machi, Takatsuki, Osaka 569-8686 Japan; 2 Department of Anesthesiology, Osaka Medical College, Osaka Medical College Hospital, Takatsuki, Osaka 569-8686 Japan

**Keywords:** Presepsin, Sepsis, Sepsis-3 definition, Inflammation-based prognostic score, Mortality

## Abstract

**Objective:**

Sepsis is a major cause of mortality for critically ill patients. This study aimed to determine whether presepsin values can predict mortality in patients with sepsis.

**Results:**

Receiver operating characteristic (ROC) curve analysis, Log-rank test, and multivariate analysis identified presepsin values and Prognostic Nutritional Index as predictors of mortality in sepsis patients. Presepsin value on Day 1 was a predictor of early mortality, i.e., death within 7 days of ICU admission; ROC curve analysis revealed an AUC of 0.84, sensitivity of 89%, and specificity of 77%; and multivariate analysis showed an OR of 1.0007, with a 95%CI of 1.0001–1.0013 (p = 0.0320).

**Supplementary Information:**

The online version contains supplementary material available at 10.1186/s13104-021-05659-9.

## Introduction

Despite adequate treatment with appropriate antibiotics, sepsis is the main cause of mortality in intensive care unit (ICU) patients. Sepsis criteria were updated in 2016 [1, 2], and sepsis is now defined as a syndrome that can lead to lethal multiorgan failure, for which an increase of ≥ 2 in the Sequential Organ Failure Assessment (SOFA) score is associated with a high in-hospital mortality rate [1]. Early goal-directed management (i.e., early recognition of severe sepsis) and early initiation of aggressive and supportive therapy are important for reducing in-hospital mortality [3, 4]. Procalcitonin (PCT) is a biomarker used for sepsis with the highest specificity and is widely adopted in clinical settings. However, PCT values can be elevated by non-septic conditions, which can in turn lead to false-positive results [5–7]. Presepsin is a subtype of soluble CD14 (CD14-ST) [8] which is released into the circulation during monocyte activation upon the recognition of lipopolysaccharide from infectious agents [9]. Presepsin values are correlated with severity of sepsis [10] and are thus potentially useful for predicting the prognosis of sepsis and monitoring the course of the disease [9, 11]. Moreover, presepsin values can be measured in less than 17 min by a chemiluminescent enzyme immunoassay (CLEIA) with a compact fully automated immunoanalyzer (PATHFAST®; Mitsubishi Chemical Medience Corporation, Tokyo, Japan) [12], which is another advantage.

Inflammation-based prognostic scores, including the Glasgow Prognostic Score (GPS; based on serum C-reactive protein (CRP) and albumin levels), neutrophil to lymphocyte ratio (NLR), platelet to lymphocyte ratio (PLR), prognostic nutritional index (PNI; based on albumin and lymphocyte counts), and the prognostic index (PI; based on serum CRP and white blood cell counts), have been adopted as significant prognostic biomarkers for several types of cancer [13]. However, no study has assessed the association of mortality with presepsin values alone or in combination with the above-mentioned inflammation-based prognostic scores in septic ICU patients. The present study aimed to test the following hypotheses: (1) presepsin can predict early and long-term mortality in sepsis patients; and (2) presepsin is superior to inflammation-based prognostic scores in predicting early/long-term mortality, and its predictive ability can be improved by combining it with inflammation-based prognostic scores.

## Materials and methods

### Patients and study design

The study design, inclusion and exclusion criteria, and definition of “inflammation presepsin scores [iPS]” used in the present study have been published previously [14]. Mortality was categorized as 28-, 60-, 90-, and 180-day mortality. Presepsin values, inflammation-based prognostic scores, iPS, and changes (Δ) in presepsin values relative to baseline values at each sampling point were compared between survivors and non-survivors.

### Laboratory assessments

Presepsin concentration was measured by CLEIA (PATHFAST®; Mitsubishi Chemical Medience Corporation, Tokyo, Japan) [12]. Assay results were obtained within 17 min. This assay can detect systemic infection (sepsis) within the range of 300 to 500 pg/ml.

### Statistical analysis

Categorical data are reported as percentages and compared using Fisher’s exact test. Continuous data are reported as medians with inter-quartile ranges and compared using the Mann–Whitney U test. ROC curves were generated for presepsin values, inflammation-based prognostic scores, iPS, and Δpresepsin, and areas under the curve (AUCs), cut-off values, sensitivities, and specificities were calculated. Presepsin values, inflammation-based prognostic scores, iPS, Δpresepsin, SOFA, and quick SOFA (qSOFA) (with P < 0.05 in univariate analysis) were examined further by multivariate logistic regression analysis for identifying potential predictors of mortality associated with sepsis. P < 0.05 was considered statistically significant. JMP software version 11.0.0 (SAS Institute Inc., NC, USA) was used for all statistical analyses.

## Results

**Fig. 1:**
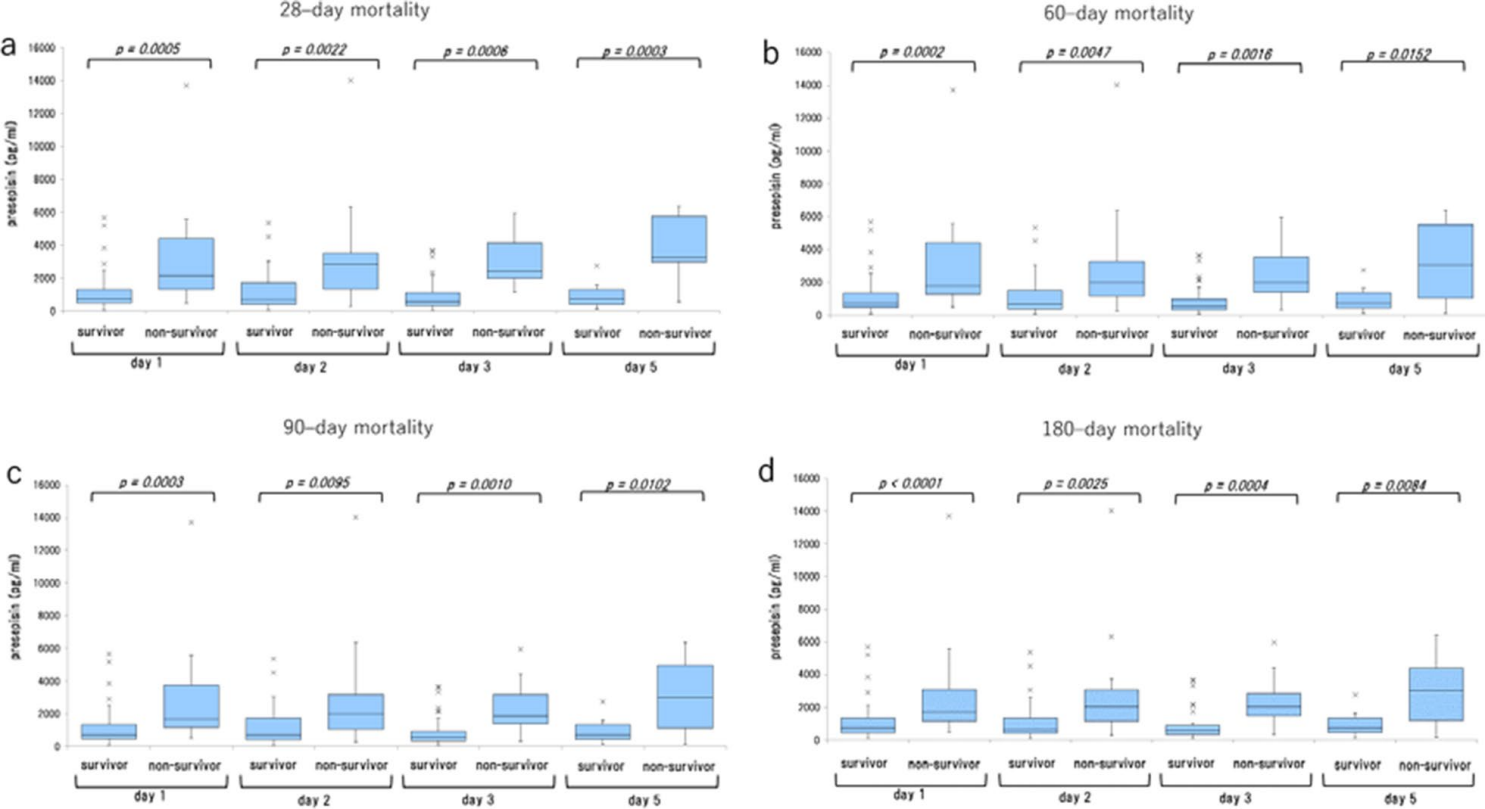
Comparison of presepsin values between sepsis survivors and non-survivors. **a** 28-day mortality, **b** 60-day mortality, **c** 90-day mortality, and **d** 180-day mortality

**Fig. 2:**
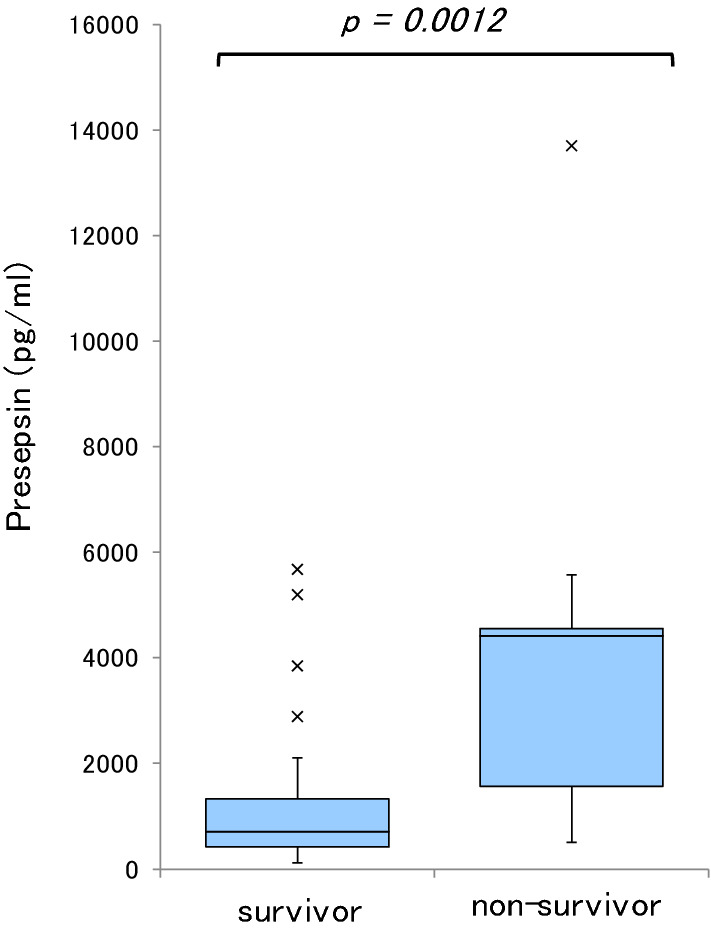
Comparison of presepsin values (Day 1) for predicting early mortality in sepsis survivors versus non-survivors

Baseline characteristics of patients are shown in Table 1. No significant differences in age or sex were observed among sepsis patients. Presepsin values of survivors were higher than those of non-survivors (Figs. 1, 2).

**Table 1:** Baseline demographic characteristics

Variable	n = 83	
Age (years)	74.0	(65.5–78.5)
Sex (male) (%)	51.0	(61.4)
Cancer (%)	40.0	(48.2)
Coronary artery disease (%)	4.0	(4.8)
Diabetes mellitus (%)	10.0	(12.0)
Hypertension (%)	21.0	(25.3)
Albumin (g/dL)	2.3	(1.8–3.0)
CRP (mg/dL)	10.4	(3.7–17.5)
WBC (× 10^9^ l^−1^)	10.9	(5.4–15.4)
Neutrophil count (× 10^9^ l^−1^)	8.7	(3.56–13.29)
Lymphocyte count (× 10^9^ l^−1^)	0.5	(0.299–0.927)
Plt count (× 10^4^ mm^−3^)	17.8	(11.5–26.5)
Fibrinogen (mg/dL)	609.0	(378–711)
Survival (dead) (%)	26.0	(31.3)
AKI (%)	38.0	(45.8)
ARDS (%)	13.0	(15.7)
Shock (%)	48.0	(57.8)
DIC (%)	30.0	(36.1)
Presepsin on Day 1 (pg/mL)	1051.5	(569–1819.3)
Presepsin on Day 2 (ng/mL)	1016.5	(538–2156)
Presepsin on Day 3 (ng/mL)	802.0	(480.5–1825)
Presepsin on Day 5 (ng/mL)	1043.0	(480–1616)
ΔPresepsin Day 2–Day 1 (pg/mL)	− 21.50	(− 246.5–274.75)
ΔPresepsin Day 3–Day 1 (pg/mL)	− 38.50	(− 748.5–304)
ΔPresepsin Day 5–Day 1 (pg/mL)	− 59.50	(− 745.75–635.5)
GPS	1.0	(1–2)
NLR	12.6	(4.53–26.35)
PLR	321.9	(195.63–543.69)
PI	1.0	(1–2)
PNI	26.6	(21.26–33.72)
SOFA	8.0	(5–11)
qSOFA	2.0	(1–3)

*CRP* C-reactive protein, *WBC* white blood cell, *AKI* acute kidney injury, *ARDS* acute respiratory distress syndrome, *DIC* disseminated intravascular coagulation, *GPS* Glasgow prognostic score, *NLR* neutrophil to lymphocyte ratio, *PLR* platelet to lymphocyte ratio, *PI* prognostic index, *PNI* prognostic nutritional index, *SOFA* sequential organ failure assessment, *qSOFA* quick sequential organ failure assessment

ROC curve analysis, Log-rank test, and multivariate analysis revealed that presepsin values and Prognostic Nutritional Index are predictors of mortality in sepsis patients (Additional file 1: Table S1, Additional file 2: Table S2, Additional file 3: Table S3, Additional file 4: Table S4). Presepsin value on Day 1 was found to be a predictor of early mortality, i.e., death within 7 days of ICU admission, with an AUC of 0.84, sensitivity of 89%, and specificity of 77% in ROC curve analysis, and an OR of 1.0007 with a 95%CI of 1.0001–1.0013 (p = 0.0320) in multivariate analysis (Additional file 5: Table S5, Additional file 6: Table S6, Additional file 7: Table S7).

## Discussion

Severe sepsis is a syndrome accompanied by lethal organ dysfunction due to dysregulated host responses to infection, and is associated with a mortality rate higher than 50% [15]. Sepsis initiates the activation of both pro- and anti-inflammatory responses [16], both of which are amplified by non-immunologic pathways including cardiovascular, neuronal, autonomic, hormonal, bioenergetic, metabolic, and coagulation pathways [17–19]. Presepsin, a subtype of CD14-ST, is secreted from granulocytes by infectious stimuli in an animal sepsis model [20] and can thus be used as a highly specific biomarker for diagnosing bacterial sepsis. Presepsin values are useful for diagnosing early stage sepsis in emergent patients and are independently associated with the degree of organ dysfunction, coagulation disorders, and ICU mortality [21, 22].

The present study demonstrated that presepsin is a predictor of mortality in septic patients. AUC values of presepsin for predicting 28-day mortality were all higher than each of the presepsin AUC values for 60-, 90-, and 180-day mortality. Moreover, multivariate analysis revealed that only the presepsin value on Day 1 predicted mortality rates. Collectively, these findings suggest that the AUC of presepsin on Day 1 might be effective in predicting early mortality (Additional file 1: Table S1, Additional file 4: Table S4).

We also investigated the ability of presepsin values on Day 1 to predict early mortality (i.e., death within 7 days of ICU admission). The AUC of presepsin on Day 1 to predict death within 7 days of ICU admission was 0.84 (Additional file 5: Table S5). Multivariate logistic regression analysis for mortality found the presepsin value on Day 1 to be a significant independent predictor of death within 7 days of ICU admission (OR, 1.0007; 95%CI, 1.0001–1.0013; p = 0.032; Additional file 6: Table S6). Ulla et al. reported that a higher level of presepsin at presentation in the emergency department was correlated to 60-day mortality [23]. Masson et al. found that presepsin levels measured at the time of presentation among patients with severe sepsis or septic shock were higher in non-survivors than in survivors [24], and our findings were consistent with these results.

In the present study, presepsin cut-off values for predicting each designation of mortality were higher than previously reported cut-offs for diagnosing either sepsis (300 to 500 pg/ml) or severe sepsis (500 to 1000 pg/ml) [25, 26]. Klouche reported that a cut-off presepsin value for mortality in sepsis patients was 1926 pg/ml [27]. These results suggest the need to adopt a higher presepsin cut-off value for predicting mortality associated with sepsis.

The presepsin values for ΔDay 3-Day 1 in 28- or 180-day mortality showed higher specificity for predicting 28- or 180-day mortality compared with presepsin values on Days 1, 2, 3, and 5 alone for all mortality (Additional file 1: Table S1). These findings suggest that Δpresepsin Day 3-Day 1 may be useful as a “rule in” test for predicting mortality. Log-rank test also revealed Δpresepsin Day 3-Day 1 and Δpresepsin Day 2-Day 1 to be independent predictors of mortality in septic patients (Additional file 2: Table S2). Presepsin values on Days 3 and 5 had higher AUCs for predicting 28-day mortality than the presepsin values on Days 1 and 2 (Additional file 1: Table S1). These results suggest the importance not only of measuring presepsin values at the time of ICU admission but also monitoring its changes on Day 2 and thereafter following ICU admission, in order to predict mortality in sepsis patients.

The AUC, sensitivity, and specificity of PNI for predicting 28-day mortality were 0.73, 70%, and 80%, respectively (Additional file 1: Table S1). Log-rank test revealed PNI as an independent predictor of all mortality in sepsis patients (Additional file 2: Table S2). Previously, we found that NLR is superior to other inflammation-based prognostic scores in predicting mortality among patients with gastrointestinal perforation and pneumonia [28, 29]. With regard to 28-day mortality, however, the present study found PNI to be a predictor of mortality in sepsis patients. Pinato et al. [30] reported that PNI is a predictor of poor overall survival in patients with hepatocellular carcinoma. In the Albumin Italian Outcome Sepsis trial, albumin replacement did not improve survival at 28 and 90 days among patients with severe sepsis or septic shock [31]. However, our findings suggest that, of the many variables that can be investigated for assessing inflammation, hypoalbuminemia and lymphocytopenia (albumin and lymphocyte counts used to calculate PNI) are important variables for predicting mortality in sepsis patients. In the context of predicting 28-day mortality, PNI shows a lower AUC than that of presepsin, but in clinical environments where presepsin values cannot be measured, PNI at presentation may be useful for determining 28-day mortality.

No previous study has assessed the association between presepsin values, PNI, and mortality in patients with sepsis according to the Sepsis-3 definition. Our findings are novel in this respect. Further studies will be needed to clarify how presepsin values and PNI can predict mortality in sepsis patients.

## Conclusions

Presepsin was found to be a predictor of mortality in patients with sepsis. In particular, the presepsin value on Day 1 is useful for predicting early mortality (i.e., death within 7 days of ICU admission). In addition to measuring presepsin values at the time of ICU admission, any changes in these values on Day 2 and thereafter following ICU admission should be monitored in order to predict mortality in sepsis patients. In the context of 28-day mortality, PNI was found to be a predictor for mortality in sepsis patients. Further studies aimed at understanding the exact role of presepsin values and PNI in predicting mortality in sepsis patients are warranted.

### Limitations

This study has several limitations. First, the present study was conducted in a single center with a small cohort. Second, we used a single biomarker, and comparisons were not made between presepsin and other biomarkers. Third, presepsin is elevated when renal function is reduced [32], which suggests that the diagnostic accuracy of presepsin may be influenced by declined renal function.

## Supplementary Information

**Additional file 1: Table S1.** Receiver operating characteristic curve analysis.

**Additional file 2: Table S2.** Log-rank test (all presepsin and variables for which P<0.05 are shown).

**Additional file 3: Table S3. **Predictors of mortality in sepsis patients (univariate analysis).

**Additional file 4: Table S4.** Predictors of mortality in sepsis patients (multivariate analysis).

**Additional file 5: Table S5. **Receiver operating characteristic curve analysis (Presepsin on Day 1 for predicting early or late mortality).

**Additional file 6: Table S6.** Receiver operating characteristic curve analysis (Inflammation-based prognostic scores, iPS, and SOFA for predicting early mortality).

**Additional file 7: Table S7. **Univariate and multivariate analyses for predicting mortality in sepsis patients.

## Data Availability

The datasets used and/or analyzed during the current study are available from the corresponding author on reasonable request.

## Abbreviations

GPS: Glasgow prognostic score
CRP: C-reactive protein (CRP)
NLR: Neutrophil to lymphocyte ratio
PLR: Platelet to lymphocyte ratio
PNI: Prognostic nutritional index
PI: Prognostic index
ROC: Receiver operating characteristic
AUC: Area under the curve
