# Supplementary material for: Presepsin values and prognostic nutritional index predict mortality in intensive care unit patients with sepsis: a pilot study
Source: BMC Res Notes. 2021 Jun 30;14:245. doi: 10.1186/s13104-021-05659-9 (PMC8243529; doi:10.1186/s13104-021-05659-9)
Supplement: Supplementary file 1 [file 13104_2021_5659_MOESM1_ESM.docx]

Table S1. Receiver operating characteristic curve analysis

Variable	AUC	Cut-off	P value	Sensiti[illegible]
28-day mortality				
Presepsin on Day 1 (pg/mL)	0.81	1340.00	P < 0.001	0.7[illegible]
Presepsin on Day 2 (pg/mL)	0.80	1163.00	P < 0.001	0.9[illegible]
Presepsin on Day 3 (pg/mL)	0.91	1819.00	P < 0.001	0.8[illegible]
Presepsin on Day 5 (pg/mL)	0.91	3011.00	P < 0.001	0.8[illegible]
ΔPresepsin Day 3 - Day 1 (pg/mL)	0.72	680.00	0.071	0.5[illegible]
PNI	0.73	22.73	0.001	0.6[illegible]
iPS-GPS	0.72	1.00	P < 0.001	1.0[illegible]
iPS-NLR	0.72	2.00	0.001	0.5[illegible]
iPS-PLR	0.73	1.00	P < 0.001	0.9[illegible]
60-day mortality				
Presepsin on Day 1 (pg/mL)	0.78	1340.00	P < 0.001	0.7[illegible]
Presepsin on Day 2 (pg/mL)	0.76	1163.00	0.002	0.8[illegible]
Presepsin on Day 3 (pg/mL)	0.83	1156.00	P < 0.001	0.9[illegible]
Presepsin on Day 5 (pg/mL)	0.77	3011.00	0.027	0.6[illegible]
iPS-NLR	0.71	1.00	0.001	0.8[illegible]
90-day mortality				
Presepsin on Day 1 (pg/mL)	0.76	1340.00	P < 0.001	0.6[illegible]
Presepsin on Day 2 (pg/mL)	0.73	1163.00	0.004	0.7[illegible]
Presepsin on Day 3 (pg/mL)	0.83	1156.00	P < 0.001	0.9[illegible]
Presepsin on Day 5 (pg/mL)	0.78	1399.00	0.011	0.7[illegible]
180-day mortality				
Presepsin on Day 1 (pg/mL)	0.77	1336.00	P < 0.001	0.6[illegible]
Presepsin on Day 2 (pg/mL)	0.75	1163.00	P < 0.001	0.7[illegible]
Presepsin on Day 3 (pg/mL)	0.85	1156.00	P < 0.001	0.9[illegible]
Presepsin on Day 5 (pg/mL)	0.78	1313.00	0.006	0.7[illegible]
