# Supplementary material for: Presepsin values and prognostic nutritional index predict mortality in intensive care unit patients with sepsis: a pilot study
Source: BMC Res Notes. 2021 Jun 30;14:245. doi: 10.1186/s13104-021-05659-9 (PMC8243529; doi:10.1186/s13104-021-05659-9)
Supplement: Supplementary file 2 [file 13104_2021_5659_MOESM2_ESM.docx]

Presepsin on Day 1 (pg/mL)
Presepsin on Day 2 (pg/mL)
Presepsin on Day 3 (pg/mL)
Presepsin on Day 5 (pg/mL)
ΔPresepsin Day 2 - Day 1 (pg/mL)
ΔPresepsin Day 3 - Day 1 (pg/mL)
PNI
iPS-GPS
iPS-NLR
iPS-PI
iPS-PLR
60-day mortality
Presepsin on Day 1 (pg/mL)
Presepsin on Day 2 (pg/mL)
Presepsin on Day 3 (pg/mL)
Presepsin on Day 5 (pg/mL)
ΔPresepsin Day 2 - Day 1 (pg/mL)
ΔPresepsin Day 3 - Day 1 (pg/mL)
PNI
iPS-GPS
iPS-NLR
iPS-PI
iPS-PLR
90-day mortality
Presepsin on Day 1 (pg/mL)
Presepsin on Day 2 (pg/mL)
Presepsin on Day 3 (pg/mL)
Presepsin on Day 5 (pg/mL)
ΔPresepsin Day 2 - Day 1 (pg/mL)
PNI
iPS-GPS
iPS-NLR
iPS-PI
iPS-PLR
180-day mortality
Presepsin on Day 1 (pg/mL)
Presepsin on Day 2 (pg/mL)
Presepsin on Day 3 (pg/mL)
Presepsin on Day 5 (pg/mL)
ΔPresepsin Day 2 - Day 1 (pg/mL)
ΔPresepsin Day 3 - Day 1 (pg/mL)
PNI
iPS-GPS
iPS-NLR
iPS-PI
iPS-PLR

PNI, Prognostic Nutritional Index; iPS, inflamn
