# Supplementary material for: Presepsin values and prognostic nutritional index predict mortality in intensive care unit patients with sepsis: a pilot study
Source: BMC Res Notes. 2021 Jun 30;14:245. doi: 10.1186/s13104-021-05659-9 (PMC8243529; doi:10.1186/s13104-021-05659-9)
Supplement: Supplementary file 3 [file 13104_2021_5659_MOESM3_ESM.docx]

Table S3. Predictors of mortality in sepsis patients (univariate analysis)

Variable	28-day mortality (non-survivors: n= 19)	60-day mortality (non-survivors: n= 21)	90-day mortality (non-survivors: n= 23)
	P-value	*P*-value	*P*-value
Age	0.0590	0.1600	0.0679
Sex	0.7860	1.0000	1.0000
Cancer	0.4350	0.4500	0.4620
Coronary artery disease	0.2230	0.2640	0.3060
Diabetes mellitus	0.4410	0.4390	0.7190
Hypertension	0.7690	0.5680	1.0000
Albumin	0.4600	0.0115	0.0086
CRP	0.3890	0.8340	0.5550
WBC	0.6470	0.6870	0.3310
Neutrophil	0.5400	0.6710	0.3160
Lymphocytes	0.3420	0.5230	0.5790
Platelet count	0.0930	0.0749	0.0864
Fibrinogen	0.0289	0.0179	0.0186
AKI	0.0023	0.0037	0.0010
ARDS	0.0062	0.0021	0.0045
Shock	0.3190	0.1310	0.1520
DIC	0.0947	0.1860	0.4380
Presepsin on Day 1	0.0005	0.0002	0.0003
Presepsin on Day 2	0.0022	0.0047	0.0095
Presepsin on Day 3	0.0006	0.0016	0.0010
Presepsin on Day 5	0.0003	0.0152	0.0102
ΔPresepsin Day 2 - Day 1	0.3490	0.5450	1.0000
ΔPresepsin Day 3 - Day 1	0.2570	0.2680	0.1550
ΔPresepsin Day 5 - Day 1	0.4600	0.8380	0.6370
GPS	0.3429	0.6315	0.3335
NLR	0.4665	0.5843	0.3345
PLR	0.8314	0.7299	0.6609
PI	0.5762	0.8809	0.4138
PNI	0.0028	0.0083	0.0063
iPS-GPS	0.0025	0.0052	0.0041
iPS-NLR	0.0019	0.0018	0.0032
iPS-PLR	0.0010	0.0026	0.0641
iPS-PI	0.0085	0.0321	0.0257
iPS-PNI	0.7106	0.3327	0.9109
SOFA	0.5933	0.2026	0.0809
