# Supplementary material for: Presepsin values and prognostic nutritional index predict mortality in intensive care unit patients with sepsis: a pilot study
Source: BMC Res Notes. 2021 Jun 30;14:245. doi: 10.1186/s13104-021-05659-9 (PMC8243529; doi:10.1186/s13104-021-05659-9)
Supplement: Supplementary file 4 [file 13104_2021_5659_MOESM4_ESM.docx]

Table S4. Predictors of mortality in sepsis patients (multivariate analysis)

Variable	Odds ratio	95% CI	
28-day mortality			
Presepsin on Day 1	1.0025	1.00005	1.00497
60-day mortality			
Presepsin on Day 1	1.0027	1.00025	1.00508
90-day mortality			
Presepsin on Day 1	1.0027	1.00025	1.00508
180-day mortality			
Presepsin on Day 1	1.0027	1.00025	1.00508
