# Supplementary material for: Presepsin values and prognostic nutritional index predict mortality in intensive care unit patients with sepsis: a pilot study
Source: BMC Res Notes. 2021 Jun 30;14:245. doi: 10.1186/s13104-021-05659-9 (PMC8243529; doi:10.1186/s13104-021-05659-9)
Supplement: Supplementary file 5 [file 13104_2021_5659_MOESM5_ESM.docx]

Table S5. Receiver operating characteristic curve analysis (Presepsin on Day 1 for predicting early or late mortality)

Variable	AUC	Cut-off	P value	Sensitivity
Presepsin on Day 1 (pg/mL)				
Death within 7 days of ICU admission	0.84	1373.00	0.00002	0.89
Death after 7 days since ICU admission	0.71	1152.00	0.00192	0.71
