# Supplementary material for: Presepsin values and prognostic nutritional index predict mortality in intensive care unit patients with sepsis: a pilot study
Source: BMC Res Notes. 2021 Jun 30;14:245. doi: 10.1186/s13104-021-05659-9 (PMC8243529; doi:10.1186/s13104-021-05659-9)
Supplement: Supplementary file 6 [file 13104_2021_5659_MOESM6_ESM.docx]

Table S6. Receiver operating characteristic curve analysis (Inflammation-based prognostic scores, iPS, and SOFA for predicting

Variable	AUC	Cut-off	P value	Sensitivity
GPS	0.56	2.00	0.5195	0.56
NLR	0.61	37.62	0.3920	0.44
PLR	0.52	356.94	0.8889	0.56
PI	0.52	1.00	0.8319	0.89
PNI	0.70	22.73	0.0809	0.67
iPS-GPS	0.76	2.00	0.0045	0.56
iPS-NLR	0.79	2.00	0.0000	0.56
iPS-PLR	0.80	2.00	0.0000	0.44
iPS-PI	0.74	1.00	0.0000	1.00
iPS-PNI	0.59	2.00	0.3778	0.33
SOFA	0.62	9.00	0.2205	0.67
qSOFA	0.63	2.00	0.1425	0.78

AUC, area under the curve; GPS, Glasgow Prognostic Score; NLR, neutrophil to lymphocyte ratio; PLR, platelet to lymphocyt
