# Supplementary material for: Presepsin values and prognostic nutritional index predict mortality in intensive care unit patients with sepsis: a pilot study
Source: BMC Res Notes. 2021 Jun 30;14:245. doi: 10.1186/s13104-021-05659-9 (PMC8243529; doi:10.1186/s13104-021-05659-9)
Supplement: Supplementary file 7 [file 13104_2021_5659_MOESM7_ESM.docx]

Table S7. Univariate and multivariate analyses for predicting mortality in sepsis patients

	Univariate analysis	Mulitivariate analysis	
Variable	*P-value*	Odds Ratio (95% CI)	
Age	0.7014		
Sex	0.4689		
Cancer	0.2971		
Coronary artery disease	0.2559		
Diabetes mellitus	1.0000		
Hypertension	0.4358		
Albumin	0.0852		
CRP	0.8591		
WBC	0.5499		
Neutrophil	0.3952		
Lymphocytes	0.3849		
Platelet count	0.1556		
Fibrinogen	0.0011		
AKI	0.0360		
ARDS	0.0706		
Shock	0.0708		
DIC	0.2678		
Presepsin on Day 1	0.0012	1.0007	(1.0001-1.0013)
GPS	0.5366		
NLR	0.3051		
PLR	0.8792		
PI	0.8695		
PNI	0.0527		
iPS-GPS	0.0057		
iPS-NLR	0.0020		
iPS-PLR	0.0016		
iPS-PI	0.0096		
iPS-PNI	0.3112		
SOFA	0.2644		
qSOFA	0.2120		

CRP, C-reactive protein; WBC, white blood cell; AKI, acute kidney injury; ARDS, acute respiratory distr[illegible]
